# Going beyond “bad news”: A surgical case report and systematic review of the literature surrounding futile care^☆^

**DOI:** 10.1016/j.ijscr.2019.04.016

**Published:** 2019-04-28

**Authors:** Scott Hickman, Antonio Gangemi

**Affiliations:** a University of Illinois College of Medicine, United States; b University of Illinois Hospital and Health Sciences System, United States

**Keywords:** Futile care, Medical futility, Surgical futility, Case report

## Abstract

**Introduction:**

Surgeons frequently deliver “bad news” to patients, but do they know how to address situations where further surgery is considered futile? Is there a clear definition of futile care in the literature? This paper explores these questions and seeks to start a conversation about how we can train future surgeons to deliver news of futile care.

**Presentation of case:**

This paper describes how one surgical team handled a difficult case of futile care and provides an interview from the perspective of a surgical resident.

**Discussion:**

The case report gives one example of how the news of futile care was delivered and how appropriate steps were taken to provide continued management of the patient and support to the family. A systematic review of the literature surrounding futile care reveals no consensus on how to define futile care within the medical community.

**Conclusion:**

There is a paucity of information surrounding how surgeons should manage cases of futile care. The literature focuses on the physician-patient relationship and includes methods for delivering bad news, yet it fails to identify a consensus definition of futile care and does not provide guidelines that future surgeons can follow when they encounter these cases. With this paper we seek to open a discussion about how to define futile care and how to teach future surgeons best practices when managing these cases.

## Highlights

• There is a paucity of literature surrounding futile care.
• There is no consensus definition of “futile care”.
• A conversation is needed to discuss training of surgeons to manage cases of futile care.

## 1 Introduction

During the course of their careers, surgeons will encounter situations where they must weigh the benefits and burdens of certain treatments and communicate that some medical and/or surgical options may be considered futile. While the scientific literature has explored ways to deliver bad news [1], it has stopped short of clearly defining futile care and outlining best practices for management of these cases. The lack of information is notable because these types of cases account for many of the ethical and financial challenges faced by hospital systems. With an estimated incidence of futile care occurring for 3.4%–12.1% of patients [2,3], it is clear that there is a need to train physicians on how to appropriately manage these cases. This paper aims to address the scarcity of information about futile care, highlight an example of a case involving futile care, and provide a starting point for a conversation into how we can train surgeons to suitably deal with these types of cases. The work presented here has been reported in line with the SCARE criteria.

According to our review of the literature, we present the first known surgical case report to serve as an example of how a surgical team delivered news of futile care and established a comprehensive plan with the patient and family to provide continued management and support in a manner that was appropriate and conscientious of their needs. We hope that our effort will trigger a conversation at the national level about potentially establishing a formal medical training on how to manage these situations.

## 2 Presentation of case

The patient was a 49-year-old Hispanic female with a past medical history of gastric cancer status post (s/p) subtotal gastrectomy with Billroth II (gastro-jejunal anastomosis), cholecystectomy, and appendectomy who presented to our medical center emergency department with a 2-day history of persistent abdominal pain, nausea, vomiting, and poor appetite while she was visiting family living in the United States. She stated her cancer was removed in Mexico two years earlier, but she still required chemo and radiotherapy after all. Medical records from Mexico were unable to be obtained despite all medical team best efforts with both the patient and her family.

She presented with dilated intra and extra hepatic bile ducts. Attempted ERCP cannulation of the bile ducts per the gastroenterology team was unsuccessful; a patent Billroth II gastrojejunostomy was found, characterized by “congestion.” The afferent limb could not be successfully navigated to reach the papilla, likely due to adhesions.

She was then referred by the admitting medical team to interventional radiology for transhepatic cholangiogram and successful placement of percutaneous external biliary drainage to relieve biliary obstruction.

Diagnostic work up with CT scan of the abdomen showed a possible blockage of the mid transverse colon; “postsurgical changes” were also noted. Decision was made to proceed with a colonoscopy to better understand the nature of the compression of the mid transverse colon and possibly obtain endoscopic biopsies to achieve a final diagnosis.

During the colonoscopy the gastroenterology team identified an extrinsic long severe stenosis in the transverse colon, suspected due to malignant external compression. An attempt to pass the obstructed segment of colon with a colonic decompression tube resulted in colon perforation and hypertensive pneumoperitoneum leading to acute abdomen and shock. An urgent surgical consultation was then requested. After immediate needle decompression of the abdomen the patient obtained return of spontaneous circulation (ROSC) and was then urgently taken from the GI lab directly to the operating room for exploratory laparotomy.

The surgical exploration revealed ascitic fluid in the left and right paracolic gutters, as well as in the pelvis, but there was no gross fecal contamination. The entire right colon and small intestine were found to be severely distended. The stomach revealed evidence of partial gastrectomy as opposed to the previously reported subtotal gastrectomy. The walls and tissues of the afferent and efferent limb of the Billroth II as well as the gastric anastomosis felt extremely indurated and had a whitish discoloration over their wall with an overall look grossly consistent with *linitis plastica*. The anastomosis of the Billroth II was also completely stuck against the mid-transverse colon, where it seemed to be infiltrating through the wall causing extrinsic compression of the mid-transverse colon. The proximal portion of the involved mid-transverse colon was stuck against the lower margin of the left lobe of the liver, giving the impression again of an infiltrative process. The 4th portion of the duodenum had a similar whitish discoloration of its wall and similar change of consistency as the wall seemed to be inelastic and very firm. Multiple biopsies were taken of the following areas: epiploica of the transverse colon, LLQ peritoneal implants, hepatocolic ligament, periaortic lymph node, inferior margin of the left lobe of the liver, ascending colon taeniae implant, and the Billroth II anastomosis implant.

An intraoperative EGD was then performed, noting a large, exophytic mass involving the gastric mucosa of the gastrojejunal anastomosis. Two biopsies were taken at this level. After the EGD, the colon was examined for gross perforation, but none was noted. Palliative intervention was pursued, and the decision was made to perform an ascending colon colostomy to bypass the obstruction at the level of the mid-transverse colon and to place an 18 French tube gastrostomy to decompress the stomach from the impending obstruction at the level of the Billroth II anastomosis that would have prevented any further drainage of gastric contents in the near future. The patient tolerated the procedure well and did not require vasopressor support. She was taken to the surgical ICU for monitoring.

The pathology report showed findings consistent with primary gastric cancer. Evidence of poorly cohesive carcinoma was found in the biopsies of the epiploica of the transverse colon, hepatocolic ligament, periaortic lymph node, inferior margin of the left liver lobe, and the ascending colon taeniae implant. Poorly cohesive carcinoma could not be excluded from the biopsies of the peritoneal implant taken over the left lower quadrant and the Billroth II anastomosis.

### 2.1 Conversation about futile care with the patient and family

The surgical team first met with the patient and her family after the exploratory laparotomy to explain that the gross findings were significant, but that they would need to wait for the pathology report before making any further decisions. After answering any questions at that time, the team left the room so that the family could collect their thoughts. Once the pathology report returned, the surgical attending and two surgical residents (PGY-1 and PGY-4) again met with the patient and delivered the news in a manner consistent with the SPIKES protocol^1^. The attending used a Spanish interpreter to ensure accurate understanding of all this information and special consideration was given to the environment in which the news was delivered. He asked the patient who she would like in the room with her before going over her results and let her know that the team would take time to answer any questions she and her family had during the conversation. Her husband and multiple relatives were present at the meeting.

The attending took the lead and first asked the patient about her understanding of her disease, noting that she had previously been treated at another facility outside the U.S. He then asked her if she would like to hear the results of her surgery and, after obtaining her assent, explained that the findings of the surgical exploration and the results of the pathology report were consistent with a very aggressive and diffuse stage IV cancer. As the conversation unfolded, the attending paused periodically to allow the patient and her family to process the information and verbalize any feelings and questions. The family had strong emotions and the attending addressed their feelings. When they inquired about possible further intervention to remove the burden of tumor, the news was delivered that her cancer was not amenable to surgical cure and that further surgery would therefore be considered futile with regards to improving prognosis and/or quality of life. It was stressed that another surgical procedure would also carry additional risks and potential burdens.

Ultimately, after discussing the possible options for continuing her care, the patient made the decision to undergo palliative care and return to Mexico so that she could see her family. The attending surgeon consulted social work to coordinate her travel with her treatment and a hospital in Mexico was contacted so that she could have continuity of care.

### 2.2 Resident’s perspective

Six months later a phone interview was conducted with the PGY-4 surgical resident who attended the meeting and witnessed the conversation about futile care. She first remarked that she remembered much of the encounter because she “found it important and touching,” noting that conversations such as these are some of the most difficult moments in a surgeon’s career. “This can be a shocking conversation with people, or stunning, even though they might be expecting it. You’re handing someone a death sentence and they’re just like, ‘What do I do with this?’ Families are incredibly grateful for the time you spend with them. It is easy to depersonalize while operating and when you have to tell bad news, it’s very easy as a resident to say, ‘this is what it came back as,’” referring to how it can seem easier to deliver bad news just by reading off a pathology report rather than having a more nuanced discussion. She stressed the importance of the attending surgeon taking time to answer any questions the patient and her family had, noting that the patient’s husband had a tough time. “He had a lot of anger. She had been going in for abdominal pain for 2 years and he kept saying that ‘Everyone said everything was fine. How could this be? This is not fair.’ [The attending] took time to listen to him react because he needed someone to validate how scary this was for him. The patient herself was incredibly composed. She had already accepted it and kept saying she hopes the next patient finds you [the surgeon] sooner. She was just incredibly thankful. As she was being grateful for this news, I was crying as she said it. Everyone in the room was crying.”

One thing that the resident said stuck with her was the incorporation of religion into the conversation, noting that the patient came from a very religious Hispanic background. “The patient kept saying ‘God bless your hands’ and the attending talked about God with them. He went along with what her views were, which I think was appropriate, saying ‘Whatever higher power you believe in will get you through this time and yes miracles can happen, though in our experience something this advanced is not something we can do anything about except control your pain, make you comfortable and help you spend time with your family.’ He allowed himself to get sincere and emotional with the patient which I really respected. He really took the time that not every surgeon would take to spend time with the family who is stunned in the moment.”

Additionally, the resident recalled various actions that the surgical team took to create an appropriate and professional encounter. She mentioned that the team was taking notes on what issues the family needed to address with the case manager and she remarked on nonverbal parts of the conversation such as holding the patient’s hand and turning the television off so that the room was quiet. She felt that the use of an interpreter rather than an English-speaking family member was, “incredibly important even for native speakers, not only for legal protection, but to be fair to the patients. You know that someone in the family isn’t going to mistranslate or not use the medical terminology. You want to make sure there is no confusion at all and be very clear.” She also noted that when the attending used the interpreter, “he wasn’t saying ‘Hey can you ask them this?’ but he was actually having a conversation directly with the family.” The discussion lasted approximately an hour, according to the resident, and included asking the family what else they needed and providing all appropriate resources. She states that even six months after the case, she was still able to vividly recall these details because “it was pretty impactful for me. [The attending] concluded by saying we’re going to make sure you’re comfortable and surrounded by people you love. We want you to know we’ve done everything we possibly can for this illness.”

## 3 Discussion

### 3.1 Literature review

Searches were performed in electronic databases (PubMed, Scopus, and Cochrane Library) for relevant studies published between 01 January 2012 and 31 March 2018 Fig. 1. A total of 20 studies were used, including four studies from outside this time frame due to their high relevance. The search key words included the following terms: ‘futile care’, ‘medical futility’, ‘futile treatment’, ‘end of life’, ‘life sustaining’, ‘decision making’.

**Fig. 1:**
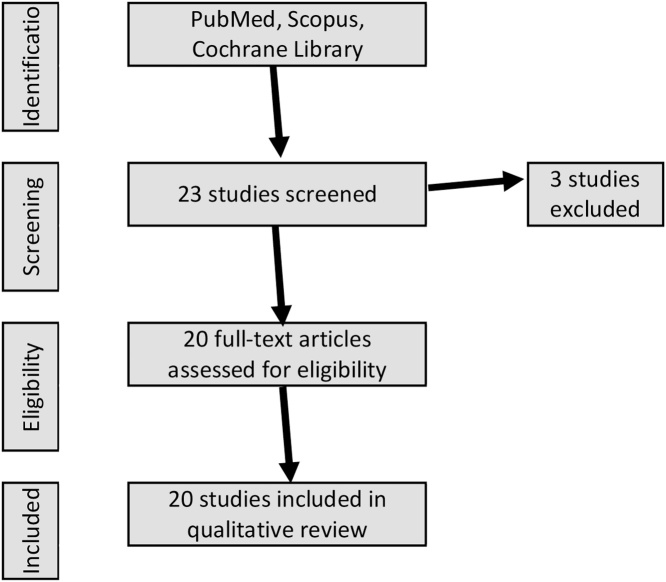
Flowchart of study selection.

Futile care is a subject that the scientific literature has struggled to define and measure, resulting in a lack of clear guidelines for physicians to follow when they encounter these cases. While it can be difficult to quantify, some studies have attempted to estimate the incidence and cost of futile care. For instance, one analysis found that 3.4% of patients met the definition of futility used in the study yet accounted for 8.9% of total costs; the costs for those who did not meet their definition of futility were found to be 2.6 times lower [2]. Another study of three tertiary hospitals in Australia looked at end-of-life admissions and found an incidence of futile treatment of 12.1% corresponding to a value of $12.4 million in Australian dollars ($9.5 million in US dollars) [3]. Although it is evident that cases of futile care occur regularly, there appears to be no consensus on how they should be addressed.

One of the reasons behind this difficulty is the lack of a universal definition of futile care across the literature. In the aforementioned study the definition of futility was restricted to patients with an ICD-9 diagnosis of injury and “*death within 7 days of discharge from a hospitalization of at least 14 days*” [2]. Others have argued that “*It is difficult to achieve a clear consensus over the concept of medical futility; hence, it should be defined and determined at an individual level and based on the unique condition of each patient*” [4]. Some have proposed treating futility as a “*factual judgment*” to refer to clinically ineffective treatments or as a “*value judgment*” to refer to situations where the benefits of treatment do not outweigh the risks or burdens [5]. Still others have proposed multiple different definitions of futile care, divided into physiological futility (“*therapy that will deliver no physiologic effect*”), qualitative futility (those that “*produce a result that may be lacking in purpose*”), and quantitative futility (“*an intervention that has a very small chance of benefiting the patient*”) [6]. While each of these proposed definitions has its utility, the lack of consensus can make it difficult for a physician to know how to proceed when he or she manages a case involving potentially futile care.

Additionally, there are factors at the physician-patient level that must be accounted for when looking at potentially futile treatments. One study looked at the relationship between physician preferences for their own end-of-life care and the amount of healthcare spending in the geographic region in which they practice, finding that those physicians in areas with higher spending tended to choose more aggressive treatment for themselves than those in areas with less spending [7]. This is particularly notable because physician treatment preferences for themselves can influence the preferences of their patients. A study looked at differences in end-of-life treatment choices between physicians and their patients and found that “*although patients and physicians as groups differ substantially in their preferences for end-of-life care, there was significant correlation between individual academic physicians' preferences and those of their primary care patients”* [8]. This reinforces the impact of the physician-patient relationship and how physicians’ own preferences can affect the decision-making of their patients.

Further supporting the importance of the physician in end-of-life care, a survey on perceived quality of life and preferences for CPR and mechanical ventilation found that “*physicians underestimate their elderly outpatients’ quality of life and that these estimations are statistically significantly correlated with physicians’ treatment preferences for patients*.” The study does note limitations including “*the validity of patients’ treatment preferences may have been diminished by their presumably limited knowledge of life-sustaining treatments and the prognoses they imply,*” but it argues that physicians should be aware that quality of life is subjective, and they should exercise caution when making decisions [9].

Given the ambiguity surrounding potentially futile treatments, it can be difficult for a provider to determine whether or not to proceed, particularly when he or she is consulted and has not been following the patient from the beginning (e.g. surgery consultation). In a review on perioperative futile care from the perspective of an anesthesiology consult, the argument is made that the physician “*should not feel compelled to participate in a procedure, which he or she believes to be contributing to futile care, or where the risks of the procedure exceed the potential benefits, especially at the end of life*” [10]. It is not advisable, however, to remove this ambiguity by creating a policy for addressing medical futility without “*collect[ing] the necessary data to identify and correct any significant harms from unilateral medical futility policies*” [11]. Rather than instituting strict policies, it may be more beneficial to embrace the ambiguous nature of futile care and end-of-life treatments in discussions with patients and their families. A randomized controlled trial in which patients in the intensive care setting were selected whether or not to receive ethics consultations found that “*There were no differences in overall mortality between the control patients and patients receiving ethics consultations. However, ethics consultations were associated with reductions in ICU hospital days and life-sustaining treatments in those patients who ultimately failed to survive to discharge. Also, ethics consultations were regarded favorably by most participants*” [12]. This reveals that open communication and discussions surrounding the ethical considerations of a particular case can lead to reduced time in the hospital and increased satisfaction of families.

Although some treatments or surgeries may be futile in a given case, the physician must be prepared to discuss other options such as palliative care and hospice and to create a comprehensive care plan. A systematic review looking at the cost and quality of care for patients pursuing palliative measures found “*evidence supported existing research that palliative care interventions generally reduce health service costs. Evidence of concurrent improvement in quality-of-life outcomes was limited*” and the review called for further research to be done [13]. Interestingly, although physicians reported wanting less treatments for themselves than patients did [8], a retrospective cohort study published in 2016 found that in the last six months of life hospitalization rates between physicians and nonphysicians were similar while “*U.S. physicians were more likely to use hospice and ICU- or CCU-level care*” [14]. This illustrates a discrepancy in physicians’ stated desires and the actual care they receive which may be indicative of the complexity of medical situations and how decision-making changes over time. This further supports the importance of open dialogue between the patient and physician throughout the course of their care so that any changes in treatment preferences can be thoroughly discussed.

While the literature has explored the physician-patient relationship and how to deliver bad news, there still remains a lack of clear training and guidelines for surgeons to manage cases involving futile care. One review found that “*families of the patients admitted to ICU value respect, compassion, empathy, communication, involvement in decision-making, pain and symptom relief, avoiding futile medical interventions, and dignified end of life care*” [15]. A 2015 policy statement adopted by the American Thoracic Society, American Association for Critical Care Nurses, American College of Chest Physicians, European Society for Intensive Care Medicine, and the Society of Critical Care Medicine recommends that “*use of the term ‘futile’ should be restricted to the rare situations in which surrogates request interventions that simply cannot accomplish their intended physiologic goal*… *the term ‘potentially inappropriate’ should be used, rather than futile, to describe treatments that have at least some chance of accomplishing the effect sought by the patient, but clinicians believe that competing ethical considerations justify not providing them*” [16]. A notable protocol for “breaking bad news,” known as the SPIKES protocol [1], is commonly used in clinical settings, but its guidelines are limited mostly to the oncological field and do not expand on how to deal specifically with futile care. Another review of the literature concluded that a multifaceted approach should be used when addressing patients and their families. “*Improved communication that begins at the start of the physician–patient relationship, the use of consultations from ethics committees, palliative care specialists, pastoral care teams, and patient representatives, as well as frank discussions with patients and families regarding the goals of care can help avoid futility conflicts and improve surgical outcomes*” [17]. An often overlooked topic during discussions surrounding goals of care is religion. “*Over 77% of surrogate decision-makers endorse religion or spirituality in one study, and yet religious/spiritual considerations were addressed in only about 16% of goals-of-care conferences, and in the majority of times only when raised by the patient's decision-makers. Physicians sought more information on the patient's religious beliefs only 3.2% of the time*” [18,19]. A qualitative study found “*A combination of strategies is necessary to reduce futile treatment, including better training for doctors who treat patients at the end of life, educating the community about the limits of medicine and the need to plan for death and dying, and structural reform at the hospital level*” [20]. The literature on futile care has identified opportunities for improvement, yet it is still an area that needs to be addressed moving forward.

The results of this literature review reveal the lack of consensus within the medical community for how to define futile care and for how it should be addressed by clinicians. Potential goals for managing cases of futile care include establishing a consensus definition of the term, evaluating current physician practices, creating guidelines, and training future professionals. We hope that our study will highlight the need for such measures and begin the conversation on how to institute these practices Table 1 .

**Table 1:** Study Characteristics.

First Author (Publication Year)	Purpose of Study	Pertinent Findings
Aghabarary (2016)	Review medical literature about how to define medical futility.	Medical futility is a complex, ambiguous, subjective, situation-specific, value-laden, and goal-dependent concept.
Baile (2000)	Create a protocol for delivering bad news.	A six-step protocol was created to address four main objectives when delivering bad news: gathering information from the patient, transmitting the medical information, providing support to the patient, and eliciting the patient's collaboration in developing a strategy or treatment plan for the future.
Bosslet (2015)	Provide recommendations to prevent and manage intractable disagreements about the use of treatments that clinicians believe should not be administered.	Use of the term "futile" should be restricted to the rare situations in which surrogates request interventions that simply cannot accomplish their intended physiologic goal. Clinicians should not provide futile interventions.
Botha (2013)	Review of the literature exploring futility of care, ethics committees, and institutional policies.	Defines futility as physiological, quantitative, or qualitative.
Carter (2017)	Estimate the incidence, duration and cost of futile treatment for end-of-life hospital admissions.	The incidence rate of futile treatment in end-of-life admissions was 12.1% across the three study hospitals (range 6.0%-19.6%). The cost associated with futile bed days was estimated to be $AA12.4 million for the three study hospitals.
Ernecoff (2015)	Determine how frequently surrogate decision makers and health care professionals discuss religious or spiritual considerations during family meetings in the intensive care unit and to characterize how health care professionals respond to such statements by surrogates.	The study found that 77.6% of surrogate decision makers reported religion or spirituality as fairly or very important, but it was only discussed with healthcare professionals in 16.1% of cases.
Fleischman (2012)	Propose a definition of futile care and quantify its cost in injured elders.	The 3.4% of patients receiving futile care incurred 8.9% of total costs.
Gallo (2017)	Determine whether physician preferences for end-of-life care were associated with variation in health care spending.	Physician preference for aggressive end-of-life care was correlated with area-level spending in the last 6 months of life.
Gramelspacher (1997)	Assess differences and correlations between physicians' and their patients' desires for end-of-life care for themselves.	Although patients and physicians as groups differ substantially in their preferences for end-of-life care, there was significant correlation between individual academic physicians' preferences and those of their primary care patients.
Grant (2014)	Discuss the definition of futility, methods for resolving futility disputes, and some ways to reframe the futility debate to a more fruitful discussion about the goals of care, better communication between surgeon and patient/surrogate, and palliative surgical care.	Suggests that improved communication and the incorporation of consultations from palliative care and ethics committees can improve surgical outcomes.
Harris (2013)	Conduct a systematic review of the evidence for palliative interventions reducing health service costs without impacting on quality of care.	Found that while there is evidence supporting the cost lowering effects of palliative care, there is insufficient evidence related to quality of life improvements.
Jox (2012)	Elucidate how clinicians define futility, when they perceive life-sustaining treatment (LST) to be futile, how they communicate this situation and why LST is sometimes continued despite being recognised as futile.	Defines futility in terms of "factual judgments" and "value judgments."
Matlock (2016)	Compare healthcare use in the last months of life between physicians and nonphysicians in the United States.	Provides preliminary evidence [that] U.S. physicians were more likely to use hospice and ICU- or CCU-level care. Hospitalization rates were similar.
Nurok (2013)	Address the question of whether perioperative futility can be defined.	Finds that there is no consensus on how to define futile care and thus there has been more of a focus on informed consent from the patient and/or family.
Rubin (2013)	Review the literature surrounding how hospital ethics committees make judgments about potentially futile treatments for patients.	Calls for more research to be done before implementing a unilateral medical futility policy.
Salins (2016)	Review the literature to determine factors influencing family satisfaction of intensive care unit care in ICU deaths.	Families of the patients admitted to ICU value respect, compassion, empathy, communication, involvement in decision-making, pain and symptom relief, avoiding futile medical interventions, and dignified end of life care.
Schneiderman (2000)	Prospective, randomized, controlled trial of ethics consultations.	There were no differences in overall mortality between the control patients and patients receiving ethics consultations. However, ethics consultations were associated with reductions in ICU hospital days and life-sustaining treatments in those patients who ultimately failed to survive to discharge. Also, ethics consultations were regarded favorably by most participants.
Uhlmann (1991)	Investigate whether perceived quality of life is associated with preferences for life-sustaining treatment for older adults.	Physicians' estimations of patient quality of life are significantly associated with physicians' attitudes toward life-sustaining treatment for the patients. For the patients, however, perceived quality of life does not appear to be associated with their preferences for life-sustaining treatment.
Van Norman (2017)	Discuss recent findings regarding what factors influence physicians and patients or their surrogates in decisions to forego life-sustaining treatments and consider whether futility arguments regarding life-sustaining treatments should be abandoned.	Physicians rarely discuss religious or spiritual beliefs with their patients.
Willmott (2016)	Investigate why doctors believe that treatment that they consider to be futile is sometimes provided at the end of a patient's life.	Doctors believe that a range of factors contribute to the provision of futile treatment. A combination of strategies is necessary to reduce futile treatment.

## 4 Conclusion

The case that has been reported is just one of many similar situations in which surgeons find themselves discussing the futility of further surgery with a patient and his or her family. The literature surrounding futile care provides minimal agreement on its definitions and makes clear that there is ambiguity when making decisions on whether to perform an intervention. Our review of the literature failed to reveal specific outlined procedures for speaking to patients and conveying their prognosis yet noted that some studies have called for a multifaceted effort to improve how futile care is handled, including better training for physicians. Ultimately, a holistic approach to futile care seems to be preferable over any singular policy or action.

Moving forward, there are many steps that can be taken to help guide current and future surgeons who encounter these types of cases. A possible strategy would be to conduct a global Delphi consensus study with the surgical community to establish a consensus definition of futile care and evaluate current physician practices. This information can then be used to craft guidelines for surgeons and a formal training can potentially be developed.

Our case serves as an example of how one surgical team delivered news of futile care and how it was perceived first-hand by a resident, yet we acknowledge that it does not represent guidelines for how to manage future cases. The case and literature review are presented to start a conversation about how doctors should be trained to handle these types of cases and the value that this training can have to reduce futile treatment and to create greater satisfaction among patients and families who feel that ethical considerations were appropriately discussed.

## Conflicts of interest

The authors have no personal or financial conflicts of interest in relation to this article.

## Sources of funding

N/A.

## Ethical approval

In its current form this case report does not require ethical approval.

## Consent

Written informed consent was obtained from the patient for publication of this case report and accompanying images. A copy of the written consent is available for review by the Editor-in-Chief of this journal on request.

## Author contribution

Dr. Antonio Gangemi – conceptualization, methodology, supervision, validation, writing – review and editing.

Mr. Scott Hickman – data curation, formal analysis, writing – original draft.

## Registration of research studies

N/A.

## Guarantor

Dr. Antonio Gangemi.
